# Validity and Reliability Study of the Turkish Version of the Transactional Analysis Scale: A Sample of Nurse Managers

**DOI:** 10.1155/jonm/6817853

**Published:** 2025-08-25

**Authors:** Ayla Tisinli, Seyda Saydamli, Merve Bat Tonkuş

**Affiliations:** Faculty of Health Sciences, Nursing Department, Istanbul Yeni Yuzyil University, Istanbul, Türkiye

**Keywords:** communication skills, nurse managers, reliability, transactional analysis, validity

## Abstract

**Aim:** This study aimed to adapt the Transactional Style Inventory for Managers (TSI-M) into Turkish and evaluate its psychometric properties among nurse managers.

**Background:** Effective communication is a core competency for nurse managers, influencing both staff satisfaction and the quality of healthcare delivery. Since communication behaviors are shaped by ego states, reflecting internalized thoughts, feelings, and experiences, a valid and reliable tool is essential to assess these states in managerial contexts.

**Methods:** The TSI-M was translated into Turkish using a standardized back-translation procedure and reviewed by a panel of experts. The sample consisted of 230 nurse managers (77% female) working in various healthcare institutions. Validity and reliability were assessed using item analysis, the content validity index (CVI), and confirmatory factor analysis (CFA).

**Results:** CFA supported the construct validity of the scale, yielding acceptable model fit indices (*χ*^2^/df = 3.11; GFI = 0.90; AGFI = 0.90; CFI = 0.94; RMSEA = 0.06; RMR = 0.06). Internal consistency was high for the total scale (Cronbach's *α* = 0.93) and good to be acceptable across subscales: parent (*α* = 0.895), adult (*α* = 0.812), and child (*α* = 0.836). While the parent and child ego state subscales demonstrated strong convergent and discriminant validity, the adult ego subscale showed slightly below-threshold composite reliability (CR = 0.692) and weaker discriminant validity.

**Conclusions:** The Turkish version of the TSI-M is a valid and reliable instrument for assessing ego states in nurse managers and holds practical value for leadership development and communication training in healthcare management.

## 1. Introduction

The rapid evolution and dynamic nature of healthcare services are pivotal factors for effective hospital management. Successful hospital management relies heavily on effective communication among healthcare teams. Communication is a fundamental component in achieving quality care, with nurse managers playing a key role in optimizing human resources, reducing staff turnover, minimizing costs, and ensuring high-quality care delivery [1]. Granados-Gámez emphasized that communication between nurses and managers directly influences employee satisfaction and the quality of care provided [2].

However, despite its importance, challenges in nurse–manager interactions persist. Kavaklı and Yıldırım found that 68% of nurses reported negative interactions with their managers, 85% felt a lack of kindness in the workplace, and 37% had normalized these negative behaviors [3]. In addition, perceptions of toxic leadership were positively correlated with increased burnout and turnover intentions among nurses [4, 5]. Workplace incivility was also linked with higher levels of psychological distress, highlighting the negative impact of poor communication on nurse well-being [6]. Therefore, enhancing communication strategies is crucial for improving job satisfaction and retention among nursing staff [7].

Unmet expectations, lack of feedback, and misunderstandings can significantly undermine nurses' professional satisfaction and organizational commitment. Moreover, ineffective communication compromises patient safety and quality of care, making its improvement a critical need in healthcare settings [7]. Conflicts between nurse managers and clinical nurses are frequently rooted in poor communication and differing interpretations, often shaped by underlying ego states. Several studies have demonstrated robust links between conflict situations and ego state dynamics [8, 9]. The communication styles of managers, shaped by personality traits and interpersonal behaviors, can significantly influence the performance and demeanor of their teams [10].

### 1.1. Role of Ego States and TSI-M

The Transactional Style Inventory for Managers (TSI-M), developed by Pareek, was used to evaluate managers' ego states. This scale categorizes ego states into three distinct types, parent, adult, and child (PAC), and examines their influence on managerial leadership and interpersonal style. Rahiman and Kodikal's study in India confirmed that the TSI-M effectively measures these ego states and demonstrates reliable factorial validity across demographic variations [11].

Subsequent research has shown that ego states significantly impact employee satisfaction and retention, especially in nursing and corporate settings [12, 13]. Recent integrative reviews indicate that nurse managers exhibiting strong adult ego traits, such as clarity, objectivity, and support, achieve better staff satisfaction, retention, and patient care outcomes [1]. Similarly, Muthuswamy and Bayomei found that leaders with stronger adult ego orientations tend to demonstrate more objective, analytical, and solution-focused leadership [14].

In light of cultural nuances and communication dynamics in Türkiye's healthcare sector, this study aimed to adapt and validate a Turkish version of the TSI-M among nurse managers. Comparable validation efforts have already been undertaken for leadership-related instruments in Turkish nursing contexts [15].

This research contributes to nursing literature by offering context-specific insights and enabling comparisons across global healthcare environments. It explores two research questions: (1) Does the Turkish TSI-M exhibit factorial validity? and (2) Do its factors demonstrate adequate internal consistency and stability?

### 1.2. Transactional Analysis (TA): A Framework for Understanding Communication and Behavior

TA, developed by Dr. Eric Berne in the 1960s, provides a model for understanding communication patterns and behaviors across various organizational contexts [16]. Originally applied in psychotherapy, TA has since been extended to fields such as psychology, education, counseling, and organizational development, offering practical tools to enhance communication skills, self-awareness, and interpersonal relationships [16, 17].

The PAC model within the TA classifies ego states into three main types. The parent ego state develops by internalizing the values, rules, and behaviors of authority figures. The adult ego state governs rational, data-driven decision-making, while the child ego state reflects emotional and spontaneous behaviors typically observed in childhood [16]. Communication between individuals is shaped by these ego states, which influence trust and understanding. Transactions, exchanges of information or behavior, can be complementary, crossed, or ulterior. For instance, an adult-to-adult transaction fosters smooth communication, whereas a parent-to-child interaction often leads to misunderstandings [17].

Several nursing studies have applied TA to explore ego states and their influence on communication and leadership in clinical and educational settings. For example, one study investigated nurse educator–student interactions in Australia, demonstrating that awareness of ego states can improve educational outcomes and reduce student anxiety [18]. Johnson et al. examined how nurse managers in the United States utilize TA to enhance leadership communication, team collaboration, and conflict resolution [18, 19]. Moreover, Özcan et al. applied TA to nurse faculty–student communication, underscoring its importance in understanding and improving leadership and communication behaviors among nurse managers [20]. These findings highlight the practical value of TA in nursing leadership and education across diverse healthcare environments.

Harris identified four core life positions that shape managerial attitudes: “I'm okay, you're okay,” “I'm okay, you're not okay,” “I'm not okay, you're okay,” and “I'm not okay, you're not okay.” Managers who adopt the “I'm okay, you're okay” position foster an environment of respect, empathy, and collaboration, which promotes positive workplace dynamics. When combined with ego states, these life positions result in four distinct interaction styles: supportive, aggressive, problem-solving, and task-obsessed [21].

Understanding ego states and transactional patterns between individuals is fundamental for facilitating effective communication and improving leadership strategies within organizations. Tools such as Pareek's TSI-M offer valuable insights into how ego states influence communication, leadership, and organizational dynamics [22]. Evaluating the applicability of this tool in the Turkish healthcare context provides a foundation for enhancing communication practices in healthcare service management.

### 1.3. Ego States and Measurement Techniques

Ego states are typically assessed through various methods, including surveys, observations, and recordings [23]. The Adjective Checklist (ACL), originally developed by Gough and Heilbrun, remains one of the most widely used and valid tools for assessing ego states across diverse cultural contexts [24]. This instrument includes five core ego state dimensions: critical parent (CP), nurturing parent (NP), adult, free child, and adapted child (AC). A recent cross-cultural study confirmed ACL's high reliability and construct validity in measuring ego states in diverse populations [25]. Pareek and Purohit refined ego state-based inventories to explore how managerial styles relate to customer orientation, innovation, and organizational performance [26].

Ego states play a significant role in shaping communication dynamics within organizations. For example, a manager operating from the CP ego state may use statements such as “This mistake should never happen again,” which can demotivate employees despite potentially producing short-term improvements [26]. In contrast, communication from the NP ego state is characterized by empathy and supportive dialog, fostering a more positive and psychologically safe work environment. When individuals communicate from the adult ego state, interactions tend to be rational, solution-focused, and directed toward problem-solving. The AC state emphasizes obedience and conformity to rules, while the natural child (NC) state encourages spontaneity and creativity [27].

Understanding ego states and their associated transactional patterns can significantly benefit organizations by promoting healthier communication practices and enabling more effective leadership strategies. Recognizing these ego state dynamics provides insight into how interpersonal relationships function within professional contexts, ultimately influencing leadership effectiveness and organizational outcomes [26].

## 2. Methods

### 2.1. Study Design, Setting, and Participants

This methodological study was conducted between July and August 2024 in both public and private hospitals in Istanbul, Turkey. Participants included nurse managers and nurses who had received training for managerial roles. The scale adaptation process began with a forward translation of the TSI-M by a bilingual translator with a background in health sciences, followed by a review by two experts in nursing and health management. A second bilingual translator, with expertise in medical linguistics, performed the back-translation into English. An expert panel of 10 professionals in nursing, psychiatry, leadership, and healthcare management evaluated each item for content validity using a four-point rating scale (1 = not suitable to 4 = definitely suitable). The content validity index (CVI) was calculated accordingly. A pilot test with 15 nurses was conducted to ensure item clarity; all confirmed that the items were understandable.

Following recommendations for confirmatory factor analysis (CFA), a minimum sample size of five participants per item was targeted. With 36 items in the TSI-M, a minimum of 180 participants was required; the final sample included 230 nurse managers; age distribution: 46% aged 31–40, 24% ≤ 30, and 30% ≥ 41; gender: 77% female; hospital type: 83% public and 18% private; departments: 49% inpatient wards, 19% outpatient clinics, 15% intensive care, 8% emergency, and 9% quality/education; managerial experience: 56% (1–5 years) and 22% (6–10 years); education level: 48% bachelor's degree; managerial roles: 55% clinical charge nurses, 34% outpatient charge nurses, and 10% quality/education units.

### 2.2. Measurements and Data Collection

Three instruments were used as follows:

1. Personal Information Form: Through this form, demographic data were collected (age, sex, experience, etc.).
2. TSI-M: Developed by Pareek and Purohit [26], this 36-item inventory assesses three ego states: parent (Items: 1, 3, 8, 10, 13, 15, 20, 22, 25, 27, 32, 34), adult (Items: 5, 12, 17, 24, 29, 36), and child (Items: remaining 18 items).
  Items are rated on a 5-point Likert scale. The TSI-M evaluates 12 managerial styles grouped into 6 bipolar pairs, based on the “OK/Not-OK” life position framework (see Appendix 1).
3. Operating effectiveness quotient (OEQ): Responses were analyzed using the OEQ matrix to determine functional and dysfunctional style interaction. The sum of each pair's scores was mapped on the OEQ matrix (see Appendix 2), yielding total scores categorized as follows: high effectiveness (> 75): balanced leadership and moderate effectiveness (50–75): developmental needs and low effectiveness (< 50): dysfunctional pattern. Ego state percentages were also calculated to identify dominant ego profiles.

### 2.3. Statistical Analysis

Data were analyzed using SPSS 22.0 and AMOS. Normality was assessed using skewness and kurtosis values. A distribution was considered acceptable if the values fell between ±1.5 [28], or more liberally ±2.0 [29, 30]. CFA was used to assess construct validity based on the theoretical model of the PAC ego states (see Appendices 1 and 2). Internal consistency was evaluated with Cronbach's alpha to determine the reliability of each ego state subscale. Convergent and discriminant validity were calculated using composite reliability (CR), average variance extracted (AVE), maximum shared variance (MSV), and average shared variance (ASV). Model fit indices were assessed using *χ*^2^/df (acceptable: 2–3; reasonable: up to 5), RMSEA, RMR, GFI, AGFI, and CFI. CFI ≥ 0.95 indicates a good fit; 0.90–0.94 indicates an acceptable fit. RMR < 0.05 indicates a good fit; 0.06–0.08 indicates an acceptable fit [31, 32]. Internal consistency was assessed with Cronbach's alpha to evaluate the reliability of each ego state subscale. Convergent and discriminant validity were calculated using CR, AVE, MSV, and ASV.

## 3. Results

Following the validation of the model through CFA, the OEQ was used to further evaluate participants' managerial style profiles and ego state balance. OEQ scores were calculated based on the sum of each “OK” and “Not-OK” style pairing using Pareek's standardized matrix (see Appendix 2) [32]. These scores were interpreted using norm-based thresholds (see OEQ Norms; Pages 269–277), allowing participants to be categorized into five effectiveness levels: very low, low, average, high, and very high.

Table 1 summarizes the demographic and professional characteristics of the 230 participating nurse managers.

**Figure 1:**
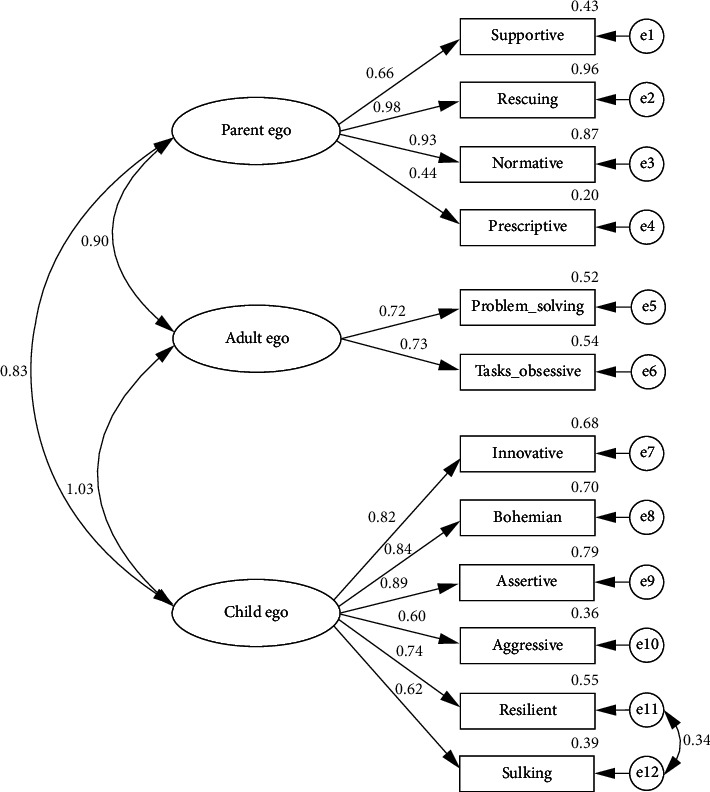
The Türkiye version of the 36-item ego state analysis instrument with factor loadings and interfactor correlations (*N* = 230). *Note:* This confirmatory factor analysis (CFA) model is based on the original TSI-M developed by Pareek and Purohit [23]. Items are grouped under three rescuing normative groups. Standardized factor loadings are shown on the paths from latent variables to observed items. Double-headed arrows represent interfactor correlations.

In this study, aiming to determine the psychometric properties of the TSI-M, CFA was performed to assess construct validity. CFA is a commonly used method for validating scales adapted for different cultures. It was employed to verify the validity of the predetermined structure [33]. Fit indices (GFI, CFI, AGFI, RMR, and RMSEA) and path diagrams obtained from the CFA (Figure 1) were analyzed.

According to the results of the CFA, the item factor loadings ranged from 0.44 to 0.98. These findings indicate that each factor established a statistically significant relationship with different variables (*p* < 0.001) [32] (see Appendix 4).

The parent ego factor most strongly explained the “Supportive” variable (standardized regression weight = 0.659). High correlations were also observed with “Rescuing” (0.978) and “Normative” (0.932), whereas the “Prescriptive” variable showed relatively lower explanatory power (0.444). The adult ego factor demonstrated similar influence on both the “Problem-Solving” (0.720) and “Task-Obsessive” (0.734) variables. The child ego factor showed strong explanatory power for “Innovative” (0.824), “Bohemian” (0.836), and “Assertive” (0.888), while still maintaining significant effects on “Aggressive” (0.598), “Sulking” (0.625), and “Resilient” (0.738) (see Figure 1).

The *χ*^2^/df ratio was calculated as 3.11, indicating an acceptable model fit. Fit indices were as follows: GFI = 0.90, AGFI = 0.90, CFI = 0.94, RMSEA = 0.06, and RMR = 0.06. These values suggest sufficient alignment between the model and observed data, supporting the construct validity of the Turkish adaptation of the TSI-M (see Table 2) [34].

Internal consistency was assessed using Cronbach's alpha. The results indicated very high internal consistency for the total scale (*α* = 0.930) and good reliability for the parent ego (*α* = 0.895), adult ego (*α* = 0.812), and child ego (*α* = 0.836) subscales (see Table 3) These results meet widely accepted thresholds for internal consistency (≥ 0.90 = very high; 0.80–0.89 = good), as suggested by de Oliveira et al. [33].

Item–total correlation analysis revealed that all items had strong positive correlations with the overall scale. Notably, “Rescuing” (0.826) and “Assertive” (0.831) items showed high item–total correlations, indicating strong discriminatory power [35].

Internal consistency was assessed using Cronbach's alpha. The results indicated excellent reliability for the total scale (*α* = 0.930) and good reliability for the parent ego (*α* = 0.895), adult ego (*α* = 0.812), and child ego (*α* = 0.836) subscales [32]. Item–total correlation analysis revealed that all items had strong positive correlations with the overall scale. Notably, “Rescuing” (0.826) and “Assertive” (0.831) items showed high item–total correlations, indicating strong discriminatory power [32].

Convergent and discriminant validity were examined for each ego state. The parent ego demonstrated strong internal consistency (CR = 0.854; AVE = 0.614) and adequate discriminant validity (MSV = 0.334; ASV = 0.211). The child ego also showed high reliability (CR = 0.889) and convergent validity (AVE = 0.577), with sufficient discriminant validity (MSV = 0.398; ASV = 0.244). In contrast, the adult ego displayed weaker psychometric indicators. While the AVE value of 0.529 was acceptable, the CR (CR = 0.689) was slightly below the recommended threshold [32–35]. Discriminant validity was also marginal (MSV = 0.398; ASV = 0.232). These results suggest that while the adult ego construct is statistically valid, it may require refinement in future cultural adaptations.

The item–total statistics after removing inappropriate items are presented in Table 4. The analysis indicated that all retained items demonstrated acceptable item–total correlation values (> 0.40), and Cronbach's alpha values increased when inappropriate items were removed, indicating improved internal consistency.

In this context, the validity indices for the PAC ego structures provided insights into the reliability and validity of the scales.  Table 5 lists the validity and reliability indices.

The CR for the parent ego factor was 0.861, and the AVE was 0.616. The square root of the AVE (0.785) indicated a high level of internal consistency. The MSV for the parent ego construct was 0.336, whereas the ASV was 0.228. A CR value exceeding 0.70 provides robust empirical evidence for the scale's reliability [32]. These findings suggest that the parent ego construct is sufficiently distinct from the other constructs and shows a high degree of discriminant validity according to modern evaluation standards [35].

The CR of the adult ego construct was calculated as 0.719, falling slightly below the widely accepted cutoff value of 0.70 [35]. The AVE was 0.537, marginally surpassing the 0.50 threshold, indicating acceptable but limited convergent validity [32]. These findings point to borderline internal consistency for the adult ego dimension. A CR value below 0.70 suggests that the related items may not consistently measure the underlying construct [35].

One possible explanation may lie in cultural differences. In the Turkish context, characteristics such as objectivity and emotional neutrality, central to the adult ego state, may be less clearly distinguished or expressed, potentially lowering item intercorrelations and weakening construct validity [35]. To improve measurement precision, future studies are encouraged to culturally adapt or revise the scale items.

While the square root of the AVE (0.687) supports convergent validity [32], the MSV (0.398) and ASV ( 0.236) suggest that discriminant validity was marginal [35, 36]. Overall, these results indicate that the adult ego construct shows lower reliability and discriminant validity compared to other subscales. While the overall reliability of the scale was supported, the adult ego factor exhibited comparatively weaker psychometric performance (CR = 0.703; AVE = 0.472) [37]. This may reflect cultural and contextual influences. In the Turkish healthcare context, organizational hierarchies, collectivist norms, and strong emotional expressiveness may obscure the distinct traits of the adult ego, such as analytical reasoning, objectivity, and emotional neutrality [38]. Although essential for reflective leadership and effective problem-solving, these qualities may not be clearly encouraged or differentiated in daily managerial practice. In addition, role expectations, educational background, and leadership experience may influence how these traits are perceived and enacted. For example, nurse managers with limited experience or leadership training might rely more on directive or emotionally charged behaviors, rather than adopting the calm, data-driven communication style associated with the adult ego. These observations highlight the potential need for cultural and occupational refinement of the adult ego subscale in future adaptations of the TSI-M.

The child ego factor demonstrated strong psychometric properties, with a CR of 0.889 and an AVE of 0.577, yielding a square root of AVE of 0.759. These values indicate high internal consistency and satisfactory convergent validity [29]. Discriminant validity was also supported, as the construct's MSV (0.398) and ASV (0.244) remained below the square root of AVE. The results presented in Table 5 confirm the high reliability and validity of the parent and child ego constructs. In contrast, the adult ego construct showed comparatively weaker psychometric properties. Nonetheless, all three constructs demonstrated sufficient distinctiveness, supporting the structural validity of the scale [37].

### 3.1. Application of Ego State Profiles and OEQ Scoring

To further illustrate the utility of the TSI-M, an aggregate assessment of ego states was performed using the 12 interpersonal styles categorized under the PAC dimensions (see Table 6). These were further classified into “OK” and “Not-OK” life positions, reflecting participants' dominant communication tendencies and perceived self–other orientations, in alignment with the TA theory [39].

To further illustrate the utility of the TSI-M, an aggregate assessment of ego states was performed using the 12 interpersonal styles categorized under the PAC dimensions (see Table 6). These were further classified into “OK” and “Not-OK” life positions, reflecting participants' dominant communication tendencies and perceived self–other orientations, in alignment with the TA theory [40].

For each ego dimension, the mean scores for “OK” and “Not-OK” styles were calculated and rounded to the nearest whole number. These values were then converted into OEQ scores using Pareek's standardized OEQ matrix [40]. OEQ scores were interpreted according to five normative levels of effectiveness: very low, low, average, high, and very high (see Appendix 3 for detailed norms and scoring matrix).

### 3.2. Application Example for Defining the Ego States of Nurse Managers

The overall transactional state assessment was conducted by averaging the 12 interpersonal styles categorized under the PAC ego states, based on participants' responses (see Table 6). These ego states were further divided into “OK” and “Not-OK” subdimensions, reflecting participants' perceived life positions and behavioral tendencies, consistent with the TA theory [40].

For each ego state, the mean scores of the “OK” and “Not-OK” styles were calculated separately and rounded to the nearest whole number. These values were then transformed into OEQ scores using Pareek's standardized OEQ matrix [39].

Table 6 presents the interpersonal styles linked to each ego state and their relative dominance. Among the parent ego styles, the “OK” NP had a very low OEQ score, suggesting that supportive and empathetic leadership behaviors were not strongly adopted by participants. This aligns with findings that nurse managers in high-pressure settings, particularly those with limited formal authority and high workload, often display low empathy, one of the predictors of stress and reduced job satisfaction [39–41]. In contrast, the “OK” regulating parent style scored high (67), reflecting strong emphasis on structure, rules, and task control, traits often reinforced in clinical leadership environments [34].

For the adult ego state, the “OK” problem-solving style scored very low (35), indicating limited use of rational and analytical thinking in day-to-day management. This suggests that nurse managers may rely more on habitual or authority-based decision-making rather than reflective, analytical approaches, a pattern observed in high-stress clinical leadership roles [34].

In the child ego state, all “OK” substyles, especially the creative child, scored low (e.g., 22), pointing to restricted expression of spontaneity, innovation, and emotional flexibility in management. Meanwhile, higher scores in “Not-OK” child styles such as rebellious or emotionally reactive behaviors suggest less adaptive interpersonal tendencies, likely stemming from stress or unresolved role tensions in clinical contexts [34].

Overall, the profile indicates a leadership style dominated by control and rule-following, with weaker nurturing, analytical, and creative capacities. To foster more balanced and effective leadership, nurse managers may benefit from tailored developmental interventions that strengthen adult and NP ego states while encouraging healthier expressions of child-like creativity and resilience [34–37].

## 4. Discussion

This study aimed to evaluate the psychometric properties of the Turkish-language adaptation of the TSI-M among nurse managers. The findings indicate that the adapted scale demonstrates acceptable levels of validity and reliability within the Turkish healthcare context.

Construct validity was supported by CFA, with fit indices falling within acceptable thresholds (*χ*^2^/df = 3.11; GFI = 0.90; AGFI = 0.90; CFI = 0.94; RMSEA = 0.06; RMR = 0.06). While these indices meet the minimum adequacy criteria, they do not indicate excellent model fit and therefore should be interpreted cautiously. Standardized factor loadings ranged from 0.44 to 0.98, which were all statistically significant. These results are consistent with methodological guidelines recommending multi-index evaluation [38].

Although the overall reliability of the scale was confirmed, the adult ego subscale showed comparatively weaker CR (0.692) and marginal AVE ( 0.529). These values fall slightly below commonly accepted thresholds, indicating that the items within this subscale may not converge strongly on a single latent construct. One plausible explanation lies in cultural interpretation differences. In Turkish society, where collectivism, emotional expressiveness, and deference to authority are prevalent, traits typically associated with the adult ego, such as rationality, objectivity, and emotional neutrality, may be less distinctly recognized or socially reinforced [41, 42]. Similar challenges have been observed in the Japanese context [43] and in studies on managerial stress [44]. In addition, cultural frameworks have been identified as significant factors influencing psychological assessments [39]. These findings underscore the importance of considering cultural nuances [45] and suggest the need to refine the adult ego subscale for future adaptations.

The TSI-M scale has practical relevance in leadership development, particularly in nursing management. Beyond identifying ego states, it serves as a tool for enhancing self-awareness, improving interpersonal communication, and fostering behavioral flexibility. Nurse managers who frequently overuse the CP ego, demonstrating judgmental or authoritarian tendencies, may benefit from developing their adult ego, which emphasizes calm, data-informed, and empathetic communication [46]. Likewise, those primarily operating from the AC ego, marked by excessive compliance or hesitation, may cultivate assertiveness and autonomy through strengthening adult ego traits.

TA-based tools also play a valuable role in leadership training by improving communication dynamics, promoting team cohesion, and reducing interpersonal conflict. Leaders who can recognize and regulate their own ego state responses, and accurately interpret those of their teams, are better equipped to manage workplace challenges. Programs incorporating ego state awareness have been shown to support the development of emotionally intelligent, reflective, and adaptive leadership styles [47].

A key limitation of this study is the absence of test–retest analysis, which restricts assessment of the scale's temporal stability. Future research should incorporate longitudinal designs to evaluate consistency over time. In addition, applying the TSI-M to more diverse samples, such as nurse managers from different healthcare institutions, regions, or levels of professional experience, would improve the generalizability of the findings.

In conclusion, the Turkish adaptation of the TSI-M appears to be a psychometrically sound instrument for assessing ego states among nurse managers. Its utility extends beyond measurement, offering a practical tool for supporting leadership development and improving organizational communication. Widespread use of the TSI-M could contribute to more effective management practices and, ultimately, enhance healthcare service quality. Future studies should replicate these findings in diverse settings and explore the sensitivity of the TSI-M to leadership interventions over time.

### 4.1. Limitations

One key limitation of this study is the absence of test–retest reliability analysis, which restricts the ability to determine whether the TSI-M yields stable and consistent results over time.

Although internal consistency indicators suggested acceptable reliability, the lack of longitudinal data weakens the overall strength of the reliability evidence. This limitation stemmed from practical challenges in recontacting participants for follow-up assessment. Future studies should address this by incorporating test–retest procedures to evaluate the temporal stability of the instrument.

Another limitation concerns the homogeneity of the sample. The study was conducted among nurse managers working primarily in the public healthcare sector, which may limit the generalizability of the findings. Applying the TSI-M to more diverse managerial populations, such as those employed in private institutions or across various healthcare settings, could enhance external validity. In addition, exploring differences across demographic variables such as age, gender, and professional experience may offer further insights into the scale's sensitivity and applicability across subgroups.

## 5. Conclusions

In conclusion, the Turkish adaptation of the TSI-M appears to be a psychometrically sound instrument for assessing ego states among nurse managers. This study represents the first comprehensive adaptation and validation of the TSI-M within the Turkish healthcare context. Its utility extends beyond measurement, offering a practical tool for leadership development, enhancing interpersonal communication, and improving organizational dynamics.

While the overall structure of the scale is supported, the adult ego subscale showed comparatively lower psychometric performance. This highlights the importance of cultural sensitivity in future adaptations.

To strengthen the generalizability of the findings, future research should replicate this study in diverse healthcare settings, such as private hospitals, different regions, and among varied professional groups including physicians and allied health managers. In addition, longitudinal studies are recommended to assess the test–retest reliability and to evaluate the sensitivity of the TSI-M to leadership development interventions over time.

Broad use of the TSI-M may ultimately support the cultivation of emotionally intelligent, balanced, and adaptive leadership styles, contributing to more effective healthcare management and improved service quality.

## Figures and Tables

**Table 1:** Distribution of nurse managers: sociodemographic and work traits (*N* = 230).

Characteristics	*n*	%
Age (years)		
< 30	50	24
31–40	98	46
≥ 41	64	30
Sex		
Female	163	77
Male	64	23
Qualification		
Registered nurse (RN)	102	48
Bachelor's degree	26	12
Nursing diploma	23	11
Marital status		
Married	148	70
Single	64	30
Type of hospital		
Public	175	83
Private	37	17
Department type		
Emergency services	17	8
Intensive care units	31	15
Patient care services	103	44.8
Polyclinics	41	19
Quality/training services	20	8.7
Years of experience		
1–5 years	19	9
6–10 years	73	31.7
11–15 years	58	27
16–20 years	34	16
21 years and older	28	12.2
Experience in a managerial position		
Less than 1 year	28	13
1–5 years	119	56.1
6–10 years	48	22
11 years and older	17	8

**Table 2:** Model fit indices for the transactional style inventory (TSI-M) (*N* = 230).

Index	Good fit	Acceptable fit	Obtained value	Outcome
*χ* ^2^/df	0 ≤ *χ*^2^/df ≤ 3	3 ≤ *χ*^2^/df ≤ 5	3.11	Acceptable
GFI	0.95 ≤ GFI ≤ 1.00	0.90 ≤ GFI < 0.95	0.90	Acceptable
AGFI	0.90 ≤ AGFI < 1.00	0.85 ≤ AGFI < 0.90	0.90	Good
CFI	0.97 ≤ CFI ≤ 1.00	0.90 ≤ CFI < 0.97	0.94	Acceptable
RMR	0 ≤ RMR < 0.05	0.05 ≤ RMR < 0.08	0.06	Acceptable
RMSEA	0 ≤ RMSEA < 0.05	0.05 ≤ RMSEA < 0.08	0.06	Acceptable

*Note:* Values meet acceptable thresholds. AGFI indicates good fit; all other indices suggest an acceptable model fit based on established cutoffs. Chi-square/df (*χ*^2^/df).

Abbreviations: AGFI, adjusted goodness of fit index; CFI, comparative fit index; GFI, goodness of fit index; RMSEA, root mean square error of approximation; RMR, root mean square residual.

**Table 3:** Internal reliability coefficients of the TSI-M scale.

Variables	Cronbach's α	Number of items
Ego state	0.930	36
Parent ego	0.895	12
Adult ego	0.812	6
Child ego	0.836	18

*Note:* Internal consistency was assessed using Cronbach's alpha (α) coefficients. Values above 0.7 are generally considered acceptable, while values above 0.80 indicate good reliability. The coefficients presented reflect the consistency of each ego state subscale, parent, adult, and child.

**Table 4:** Item–total statistics after removing inappropriate items.

Items	If item deleted, scale mean	If item deleted, scale variance	Item–total correlation	If item deleted, Cronbach's α
Supportive	18.6942	77.072	0.625	0.927
Rescuing	18.6359	72.496	0.826	0.919
Normative	18.7087	73.671	0.782	0.921
Prescriptive	18.2621	77.589	0.481	0.934
Problem-solving	18.7816	74.630	0.719	0.924
Task-obsessive	18.5534	73.683	0.751	0.923
Innovative	18.6553	74.998	0.760	0.922
Bohemian	18.7913	75.434	0.780	0.922
Assertive	18.7330	74.509	0.831	0.920
Aggressive	18.9078	80.035	0.562	0.929
Resilient	18.5922	73.199	0.737	0.923
Sulking	18.2621	73.902	0.619	0.929

**Table 5:** Convergent validity and discriminant validity of the TSI-M Scale.

EGO	CR	AVE	√AVE	MSV	ASV
Parent	0.861	0.616	0.785	0.336	0.228
Adult	0.719	0.537	0.732	0.336	0.260
Child	0.893	0.579	0.761	0.336	0.244

Abbreviations: ASV, average shared variance; AVE, average variance extracted; CR, composite reliability; MSV, maximum squared variance.

**Table 6:** Distribution of ego states, interpersonal styles, and OEQ scores.

Ego state	Interpersonal style	*N*	Mean	OEQ score	Ego state level
Parent	“Okay” nurturing parent	230	5	33	Very low
	“Not okay” nurturing	230	7		
	“Okay” regulating parent	230	5	67	Very high
	“Not okay” regulating parent	230	4		

Adult	“Okay” adult	230	5	28	Very low
	“Not okay” adult	230	8		

Child	“Okay” creative child	230	5	22	Very low
	“Not okay” creative child	230	10		
	“Okay” reactive child	230	7	36	
	“Not okay” reactive child	230	10		
	“Okay” adaptive child	230	5		
	“Not okay” adaptive child	230	12	18	

*Note:* Higher scores reflect more effective expression of the corresponding ego state.

Abbreviation: OEQ = operating effectiveness quotient.

## Data Availability

The datasets generated and/or analyzed during the current study are available from the corresponding author upon reasonable request.

## Supporting Information

The supporting appendix includes four sections. Appendix 1 presents the managerial styles and scoring framework of the TSI-M. Appendix 2 provides the OEQ scoring matrix. Appendix 3 outlines the norms and interpretation guidelines for OEQ scores. Appendix 4 reports the standardized factor loadings of TSI-M items by subscale, based on CFA.
