# Supplementary material for: Validity and Reliability Study of the Turkish Version of the Transactional Analysis Scale: A Sample of Nurse Managers
Source: J Nurs Manag. 2025 Aug 25;2025:6817853. doi: 10.1155/jonm/6817853 (PMC12401603; doi:10.1155/jonm/6817853)
Supplement: Supporting Information — Additional supporting information can be found online in the Supporting Information section. [file 6817853.f1.docx]

**APPENDIX**

**Appendix 1:** Managerial Styles and Scoring Framework (TSI-M)

| **No.** | **Managerial Style** | **Ego State Function** | **Statements (Item Numbers)** | **Score Total** |
| --- | --- | --- | --- | --- |
| 1 | Supportive | OK nurturing Parent | 1, 13, 25 |  |
| 2 | Rescuing | Not-OK nurturing Parent | 8, 20, 32 |  |
| 3 | Normative | OK regulating Parent | 3, 15, 27 |  |
| 4 | Prescriptive | Not-OK regulating Parent | 10, 22, 34 |  |
| 5 | Problem-solving | OK Adult | 5, 17, 29 |  |
| 6 | Task-obsessive | Not-OK Adult | 12, 24, 36 |  |
| 7 | Innovative | OK creative Child | 11, 23, 35 |  |
| 8 | Bohemian | Not-OK creative Child | 6, 18, 30 |  |
| 9 | Resilient | OK adaptive Child | 7, 19, 31 |  |
| 10 | Sulking | Not-OK adaptive Child | 2, 14, 26 |  |
| 11 | Assertive | OK reactive Child | 9, 21, 33 |  |
| 12 | Aggressive | Not-OK reactive Child | 4, 16, 28 |  |

Source: From Leadership and strategic management for TB control managers: Module 8, Managerial styles (p. 12), by World Health Organization, Regional Office for South-East Asia, 2008, World Health Organization.

Note: This appendix presents the 12 managerial styles included in the TSI-M, grouped under six pairs. Each style is measured by three items rated on a 5-point Likert scale. Styles are further classified as reflecting either an “OK” or “Not OK” ego state, based on Transactional Analysis theory. These classifications are used in OEQ calculations and interpretation.

**Appendix 2.** Operating Effectiveness Quotient (O E Q **)**

**OK scores**

|  |  | 3 | 4 | 5 | 6 | 7 | 8 | 9 | 10 | 11 | 12 | 13 | 14 | 15 |
| --- | --- | --- | --- | --- | --- | --- | --- | --- | --- | --- | --- | --- | --- | --- |
| **N** | 3 | 0 | 100 | 100 | 100 | 100 | 100 | 100 | 100 | 100 | 100 | 100 | 100 | 100 |
| **o** | 4 | 0 | 50 | 67 | 75 | 80 | 83 | 85 | 87 | 89 | 90 | 91 | 92 | 92 |
| **t** | 5 | 0 | 33 | 50 | 60 | 67 | 71 | 75 | 78 | 80 | 82 | 83 | 85 | 86 |
|  | 6 | 0 | 25 | 40 | 50 | 57 | 62 | 67 | 70 | 73 | 75 | 77 | 78 | 80 |
| **O** | 7 | 0 | 20 | 33 | 43 | 50 | 55 | 60 | 64 | 67 | 69 | 71 | 73 | 75 |
| **K** | 8 | 0 | 17 | 28 | 37 | 44 | 50 | 54 | 58 | 61 | 64 | 67 | 69 | 70 |
|  | 9 | 0 | 14 | 25 | 33 | 40 | 45 | 50 | 54 | 57 | 60 | 62 | 65 | 67 |
| **S** | 10 | 0 | 12 | 22 | 30 | 36 | 42 | 46 | 50 | 53 | 56 | 59 | 61 | 63 |
| **c** | 11 | 0 | 11 | 20 | 28 | 33 | 38 | 43 | 47 | 49 | 53 | 55 | 58 | 60 |
| **o** | 12 | 0 | 10 | 18 | 25 | 31 | 36 | 40 | 44 | 47 | 50 | 53 | 55 | 57 |
| **r** | 13 | 0 | 9 | 17 | 23 | 28 | 33 | 37 | 41 | 44 | 47 | 50 | 52 | 54 |
| **e** | 14 | 0 | 8 | 15 | 21 | 27 | 31 | 35 | 39 | 42 | 45 | 48 | 50 | 52 |
| **s** | 15 | 0 | 8 | 14 | 20 | 25 | 29 | 33 | 37 | 40 | 43 | 45 | 48 | 50 |

Source: Pareek and Purohit (2018), as cited in Rahiman and Kodiman (2020).

**Appendix 3.** Norms and Interpretation of Operating Effectiveness Quotient (OEQ)

Source: Pareek and Purohit (2018).

| **Function** | **Very Low** | **Low** | **Average** | **High** | **Very High** |
| --- | --- | --- | --- | --- | --- |
| Nurturing | Below 38 | 39–45 | 46–54 | 55–62 | >62 |
| Regulating | Below 35 | 36–45 | 46–55 | 56–65 | >65 |
| Managerial | Below 43 | 44–50 | 51–55 | 56–67 | >67 |
| Creative | Below 50 | 51–60 | 61–67 | 68–84 | >84 |
| Reactive | Below 45 | 46–55 | 56–62 | 63–80 | >80 |
| Adaptive | Below 47 | 48–60 | 61–68 | 69–80 | >80 |

Source: Pareek and Purohit (2018), as cited in Rahiman and Kodiman (2020).

Note:This appendix presents the standardized factor loadings of each item on the respective subscales, derived from confirmatory factor analysis.

**Appendix 4.** TSI-M by Subscales and Standardized Factor Loadings

| **Item Code** | **Subscale** | **Standardized Factor Loading** |
| --- | --- | --- |
| PR1 | Prescriptive (Parent) | 0,44 |
| NR1 | Normative (Parent) | 0,93 |
| RS1 | Rescuing (Parent) | 0,98 |
| PT1 | Supportive (Parent) | 0,66 |
| PS1 | Problem-Solving (Adult) | 0,72 |
| TO1 | Task-Obsessive (Adult) | 0,73 |
| AS1 | Assertive (Child) | 0,89 |
| BO1 | Bohemian (Child) | 0,84 |
| IN1 | Innovative (Child) | 0,82 |
| CL1 | Aggressive (Child) | 0,6 |
| SP1 | Sulking (Child) | 0,62 |
| EX1 | Resillient (Child) | 0,74 |

Note: This table presents the factor loadings of each TSI-M item by subscale, based on the CFA results conducted in this study. Factor loadings above 0.40 are generally considered acceptable, indicating a sufficient contribution of each item to the corresponding latent construct.
